# Behavioral tests assessing neuropsychiatric phenotypes in adolescent mice reveal strain- and sex-specific effects

**DOI:** 10.1038/s41598-020-67758-0

**Published:** 2020-07-09

**Authors:** Ahmed Eltokhi, Barbara Kurpiers, Claudia Pitzer

**Affiliations:** 1 Department of Neurology and Epileptology, Hertie Institute for Clinical Brain Research, University of Tübingen, Tübingen, Germany; 2 Interdisciplinary Neurobehavioral Core, Heidelberg University, Heidelberg, Germany

**Keywords:** Social behaviour, Neuroscience, Learning and memory

## Abstract

In humans, infancy and adolescence are associated with major changes in synaptic functions and ongoing maturation of neural networks, which underlie the major behavioral changes during these periods. Among adult cases with neuropsychiatric disorders including autism spectrum disorder, schizophrenia, attention deficit hyperactivity, and bipolar disorders, 50% have developed behavioral symptoms and received a diagnosis before 15 years of age. However, most of the behavioral studies in mice modeling neuropsychiatric phenotypes are performed in adult animals, missing valuable phenotypic information related to the effect of synaptic maturation during development. Here, we explored which behavioral experiments assessing neuropsychiatric phenotypes can be performed during a specific window of development in adolescent male and female C57BL/6N, DBA/2, and FVB/N mice that are typically used as background strains for generating genetically-modified mouse models. The three wild-type strains were evaluated across anxiety, social behaviors, and cognitive functions in order to cover the main behavioral impairments that occur in neuropsychiatric disorders. During adolescence, the three strains displayed significant differences under certain behavioral paradigms. In addition, C57BL/6N and FVB/N, but not DBA/2 mice revealed some sex-related differences. Our results provide new insights into discrete behaviors during development and emphasize the crucial importance of the genetic background, sex, and experimental settings in the age-dependent regulation of different behaviors.

## Introduction

Over the last 30 years, many genetically-engineered mouse lines have been generated to model human genetic disorders including neuropsychiatric disorders. Mice exhibit approximately 90% gene homology with humans^1^, and the protein-coding regions of the mouse and human genomes are on average to 85% identical^2^, supporting their high validity to study the molecular and behavioral changes involved in the pathogenesis of neurological disorders. As behavior represents the final output of the CNS and is used for the evaluation of novel drugs or the effect of genetic modifications, functional genetic analyses in mice modeling neuropsychiatric disorders rely on the efficient and in-depth characterization of the behavioral spectrum^3^. Investigation of the behavioral phenotypes of mutant mice has established and characterized mouse models of neuropsychiatric disorders including autism spectrum disorder (ASD)^4–8^, schizophrenia (SZ)^9–11^, attention deficit hyperactivity disorder (ADHD)^12,13^ and bipolar disorder (BD)^14,15^. Moreover, it contributed to our understanding of genetic and molecular mechanisms underlying complex behaviors, such as circadian rhythm, anxiety, and cognitive functions^16^. This progress is based on establishing a variety of assays for mouse behavioral phenotyping which has been developed over the years to maximize the scope and reproducibility of findings, minimize artifacts and false-positive results and provide robust and valid translational tools for testing hypotheses and developing novel treatments^17,18^.

Behavioral tests in mice are mostly burdened by inherent complexity and require consideration of several aspects such as sources of variability and experimental interference that could preclude spontaneous behavior^19^. The choice of the behavioral test is another important issue, and several tests exist for different behavioral categories covering a wide range of neuropsychiatric symptoms^18^. Furthermore, recapitulating the behavioral features of a human disease is a prerequisite for the translation of preclinical results into clinical applications^20^. One additional component to ensure the test validity for modeling neuropsychiatric disorders is behavioral experiments on mutant mice during a life stage similar to the onset of the disorder in humans. This demand derives from similar patterns of disease pathogenesis and systemic physiology in humans and mice^21^ despite the large difference in lifespan.

The onset of many neuropsychiatric disorders including ASD, SZ, ADHD, and BD emerge mainly during infancy and adolescence^22^. Thus, 73.9% and 50% of adults with neuropsychiatric disorders received a diagnosis before 18 and 15 years of age, respectively^23^. However, behavioral studies modeling neuropsychiatric disorders are mostly performed in adult mutant mice. Although working with adult mice is advantageous for easy handling, measuring social interaction during mating, and assessing complex behavioral and cognitive abilities, valuable information on the impact of synaptic maturation on the behavior during development is missed. Previously, we have shown that behavioral results including ultrasonic vocalizations (USV) are sensitive to small developmental progress even in wild-type strains^24^. Moreover, it has been shown that young- and middle-aged mice exhibited different behavioral patterns, and old-aged mice showed a decreased locomotor activity and increased anxiety‐like behavior compared to those young and middle‐aged mice^25^. Therefore, electrophysiological recordings that are mainly performed in infant and adolescent mice cannot be directly correlated to the results of the behavioral experiments in adulthood.

In this study, we aimed to investigate which behavioral tests recapitulating neuropsychiatric symptoms can be performed on adolescent mice till P40. As the prevalence, age of onset, and clinical symptoms of many neuropsychiatric disorders substantially differ between males and females (male-biased: ASD and ADHD, female-biased: depression and anxiety disorders^26^), experiments were performed on both sexes to investigate whether males and females differ at this young age. For the behavioral phenotyping, we employed a broad test battery including a comprehensive standardized methodological approach assessing behaviors in a home-cage-like environment, as well as assays covering social, sensory, and cognitive abilities. As the genetic background influences behavioral characteristics^27^, we performed our experiments on three different inbred mouse strains, C57BL/6N, DBA/2, and FVB/N that are world-wide used in research and are the standard strains in neuroscience^24^. Inbred strains have the advantage of discerning the role of individual genes and the impact of allelic variation along with decreasing the variability^28–31^. Although the C57BL/6N strain is the background strain most frequently used for genetically-modified mice^16^, the FVB/N strain is preferable for transgenic analysis because its fertilized eggs contain large and prominent pronuclei, which facilitates the microinjection of recombinant genes^32^.

Our work indicates that during adolescence, baseline and complex behaviors differ among these commonly used mouse strains, highlighting the characteristics and potential advantages and disadvantages of the individual strain in biomedical behavioral studies.

## Results

### Innate behaviors

#### LABORAS test

**Figure 1:**
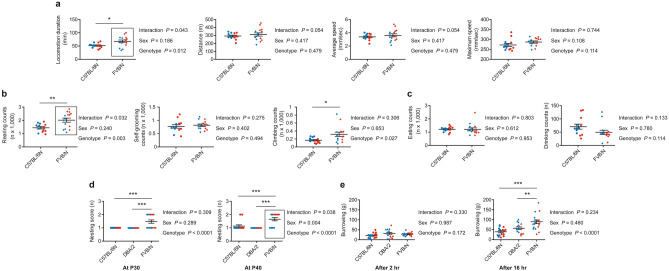
LABORAS test for C57BL/6N and FVB/N mice at P36 in which mouse movements were continuously monitored for 24 h. (**a**) High activity was observed in FVB/N mice as indicated by increased locomotion duration, traveled distance and average speed compared to C57BL/6N mice despite the similar maximum speed. (**b**) Increased repetitive rearing and climbing behaviors in FVB/N compared to C57BL/6N mice, but similar self-grooming counts. (**c**) Eating and drinking counts were similar between C57BL/6N and FVB/N mice. d) C57BL/6N and DBA/2 mice could not build nests at two different developmental stages. FVB/N mice were able to build simple nests at both developmental stages. (**e**) The burrowing test revealed a similar ability to burrow food pellets after 2 h in the three strains. However, the amount of burrowed food pellets was significantly increased in FVB/N mice compared to C57BL/6N and DBA/2 mice. Two-way ANOVA followed by Tukey post hoc test, *P ≤ 0.05, **P ≤ 0.01, ***P ≤ 0.001. A black rectangle indicates a significant difference between sexes within a strain (refer to Supplementary Table 1). Blue and red dots refer to males and females, respectively. Error bars indicate the standard error of the mean (SEM).

We investigated the behaviors of adolescent mice in a home-cage-like environment (LABORAS cages) that automatically measure the number and duration of various behavioral parameters including locomotion, eating, drinking, and repetitive behaviors. As DBA/2 mice did not reach the weight suitable for measurement in LABORAS cages before P40, we only compared C57BL/6N and FVB/N strains at P36. FVB/N mice were more active and spent more duration in locomotion compared to C57BL/6N mice (*P* = 0.012) but with similar traveled distance and average and maximum speeds (Fig. 1a). Moreover, FVB/N exhibited more repetitive behaviors such as rearing and climbing (*P* = 0.003 and 0.027, respectively) (Fig. 1b). In contrast, the numbers of eating, drinking, and self-grooming events were comparable between the two strains (Fig. 1b,c). Notably, C57BL/6N did not show any difference between male and female mice in these home-cage-like behaviors. In contrast, female FVB/N mice showed increased locomotion duration (*P* = 0.034) and rearing events (*P* = 0.035) compared to male FVB/N mice (Fig. 1a,b; Supplementary Table 1).

The LABORAS test was performed with all three mouse strains at P46 when DBA/2 mice reached a suitable weight (> 15 g). FVB/N mice still showed the highest locomotion activity, DBA/2 mice exhibited the lowest activity, and C57BL/6N mice were intermediate (Supplementary Figure 1a). Also, different from P36 mice, male FVB/N mice were more active compared to female littermates indicated by increased traveled distance (*P* = 0.016) and higher average and maximum speeds (*P* = 0.016 and 0.024, respectively) (Supplementary Figure 1a; Supplementary Table 2). For the repetitive behaviors, FVB/N mice exhibited increased repetitive rearing and climbing and decreased self-grooming compared to C57BL/6N and DBA/2 mice (Supplementary Figure 1b). Male FVB/N mice displayed significantly increased climbing counts compared to female FVB/N mice (*P* = 0.038) (Supplementary Figure 1b; Supplementary Table 2). DBA/2 mice scored decreased eating counts compared to C57BL/6N and FVB/N mice, and C57BL/6N mice obtained increased drinking counts compared to DBA/2 and FVB/N mice (Supplementary Figure 1c). Interestingly, the increased traveled distance and the higher average and maximum speed, as well as the increased climbing events in male FVB/N compared to female mice, were accompanied with a significant increase in the number of eating events in male FVB/N mice (*P* = 0.002) (Supplementary Figure 1c; Supplementary Table 2).

The 10 days difference in development during adolescence (P36 vs. P46) affected several home-cage-like behaviors. For C57BL/6N mice, the distance, average speed, and eating and drinking counts were increased with age (Supplementary Figure 2a, c). In contrast, the maximum speed of C57BL/6N mice was decreased (Supplementary Figure 2a). For FVB/N mice, the locomotion, distance, average speed, maximum speed, and eating, rearing, and climbing counts were increased in animals at P46 compared to P36 (Supplementary Figure 2a–c). The drinking and self-grooming counts of FVB/N animals were significantly decreased with age (Supplementary Figure 2b, c).

#### Nesting test

We measured the ability of C57BL/6N, DBA/2, and FVB/N mice to build nests during adolescence. Expectedly, at P30 and P40, the mice of all three strains showed a very low interest to build nests (Fig. 1d). Nonetheless, distinct to juvenile C57BL/6N and DBA/2 mice, FVB/N mice started to show this intrinsic behavior of adult mice (P30: *P* = 0.0003 vs. C57BL/6N and DBA/2; P40: *P* = 0.0001 vs. C57BL/6N and < 0.0001 vs. DBA/2) (Fig. 1d). Notably, female P40 FVB/N mice built more complex nests compared to male mice (*P* = 0.0003) (Fig. 1d; Supplementary Table 1).

#### Burrowing test

The burrowing test is another important assay to measure the innate behavior of mice and reflects the integrity of hippocampal function^33^. All three investigated strains showed a similar ability to burrow food pellets from the tube within the first 2 h. However, after 16 h, FVB/N mice burrowed significantly more food pellets than C57BL/6N (*P* < 0.0001) and DBA/2 (*P* = 0.01) mice (Fig. 1e). Burrowing behavior did not differ between male and female mice of the three strains (Supplementary Table 1).

### Anxiety-like behavior

Patients with neuropsychiatric disorders are frequently burdened by anxiety. Thus, we performed a variety of tests for measuring anxiety-like behavior in the three investigated strains, which unraveled that all of them are suited for exploration during adolescence.

#### Open field test

**Figure 2:**
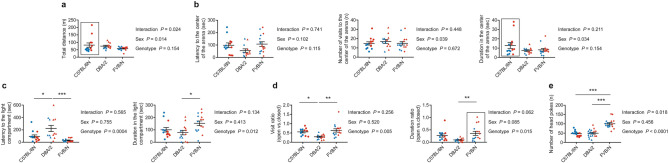
Anxiety experiments in C57BL/6N, DBA/2, and FVB/N mice. (**a**) The open field test revealed no difference in total distance in the new arena between C57BL/6N, DBA/2, and FVB/N mice. (**b**) No difference in latency, number of visits, or duration in the center of the arena between C57BL/6N, DBA/2, and FVB/N mice. (**c**) DBA/2 mice exhibited significantly higher latency to the light compartment compared to C57BL/6N and FVB/N mice and significantly lower duration compared to FVB/N in the dark/light compartment test. (**d**) DBA/2 mice showed significantly less visit ratio (open vs closed arms) compared to C57BL/6N and FVB/N mice, and significantly less duration ratio (open vs closed arms) compared to FVB/N. (**e**) Hole-board test revealed an increased count of head poke in FVB/N mice compared to C57BL/6N and DBA/2 mice. Two-way ANOVA followed by Tukey post hoc test, *P ≤ 0.05, **P ≤ 0.01, ***P ≤ 0.001. A black rectangle indicates a significant difference between sexes within a strain (refer to Supplementary Table 1). Blue and red dots refer to males and females, respectively. Error bars indicate the standard error of the mean (SEM).

The exploratory behavior and activity in the open field of all investigated mice was strain independent (Fig. 2a). Interestingly, female C57BL/6N mice were more active than males as shown by the increased total traveled distance (*P* = 0.004) (Fig. 2a; Supplementary Table 1). The latency to enter the center of the arena and the numbers of visits and duration in the center were comparable in the three strains (Fig. 2b). A significantly increased duration in the center of the arena of female C57BL/6N than male mice (*P* = 0.031) confirms their reduced anxiety (Fig. 2b; Supplementary Table 1).

#### Dark/light compartment test

Distinct to the open field test, anxiety evaluated by the dark/light compartment test differed between the strains. FVB/N mice exhibited the least anxious behavior as documented by the lowest latency to enter the light compartment (*P* = 0.0002 vs. DBA/2 and 0.306 vs. C57BL/6N) and highest duration (*P* = 0.015 vs. DBA/2 and 0.112 vs. C57BL/6N) in the light compartment (Fig. 2c). DBA/2 mice displayed the highest anxious behavior with increased latency to enter the light compartment (*P* = 0.018 vs. C57BL/6N) (Fig. 2c). Male and female mice of the investigated strains showed no difference in latency or duration of visits to the light compartment (Supplementary Table 1).

#### Elevated plus maze test

The high anxious level of DBA/2 and low anxiety of FVB/N mice were also observed in the elevated plus maze test. The relative visits of open versus closed arms were significantly less in DBA/2 than C57BL/6N (*P* = 0.032) and FVB/N (*P* = 0.003) mice (Fig. 2d), and the ratio of the duration in open versus closed arms was also significantly reduced compared to FVB/N mice (*P* = 0.007) (Fig. 2d). Female FVB/N compared to male FVB/N mice showed an increased duration ratio in open versus closed arms (*P* = 0.01), suggesting female mice to be less anxious (Fig. 2d; Supplementary Table 1).

#### Hole-board test

The hole-board test is used to examine explorative activity as an indication of anxiety-like behavior^34–36^. In line with the low anxiety in the dark/light compartment and elevated plus maze tests, FVB/N mice showed the most head pokes in the hole-board test compared to both C57BL/6N (*P* < 0.0001) and DBA/2 (*P* < 0.0001) mice (Fig. 2e). No difference between male and female mice of the investigated strains was seen in the number of head pokes (Supplementary Table 1).

### Stimulus-evoked behavior

**Figure 3:**
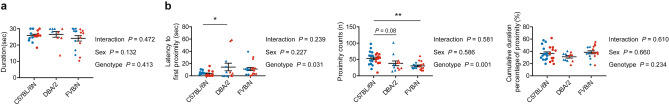
sensory and social behaviors in C57BL/6N, DBA/2, and FVB/N mice. (**a**) C57BL/6N, DBA/2, and FVB/N mice showed a similar average duration in the cold plate test. (**b**) In the direct social interaction test, C57BL/6N mice showed a decreased latency to the first proximity with a same-sex littermate mouse compared to DBA/2 and increased proximity counts compared to DBA/2 and FVB/N. The cumulative duration percentage of proximity was similar in the three strains. Two-way ANOVA followed by Tukey post hoc test, *P ≤ 0.05, **P ≤ 0.01. Blue and red dots refer to males and females, respectively. Error bars indicate the standard error of the mean (SEM).

The cold plate test is used for assessing the reaction to a cold stimulus and measuring cold hyperalgesia by the withdrawal response of one hind paw. The cold plate test was conducted with P32 mice, and the reaction to the cold temperature could be measured similarly to adult mice. The three investigated strains endured the cold temperature with similar responses (Fig. 3a). Also, no difference between male and female mice in the cold plate test was found (Supplementary Table 1).

### Social interaction

During adolescence, mice usually make social communication with other mice especially their littermates. Social interactions were measured by the latency of the first proximity, number, and cumulative duration of proximity between same-sex littermates. All three tested strains showed clear social interaction ability, and the cumulative duration of proximity was very similar (Fig. 3b). The C57BL/6N strain showed the lowest latency for the first contact with a littermate mouse (*P* = 0.041 vs. DBA/2 and 0.134 vs. FVB/N) (Fig. 3b), which was associated with the highest number of proximity counts (*P* = 0.078 vs. DBA/2 and 0.001 vs. FVB/N) (Fig. 3b). The male–female comparison within the strains revealed no significant differences in the three evaluated parameters (Supplementary Table 1).

### Cognitive function and memory

Different tests for measuring learning ability and memory function were performed on the three mouse strains during adolescence. As traditional memory tests relying on food deprivation like the T- and Y-maze or the Morris water maze tests can become life-threatening in young mice, we used the puzzle box test that includes problem-solving tasks with increasing difficulty reflecting the natural behavior of adult and adolescent mice. We also applied the natural behavior reflecting fear learning and active place avoidance tests. As the young mice showed the ability to perform these tests, we could demonstrate different learning and memory abilities or, at least, different levels of handling the experiments in the three strains during adolescence.

#### Puzzle box test

**Figure 4:**
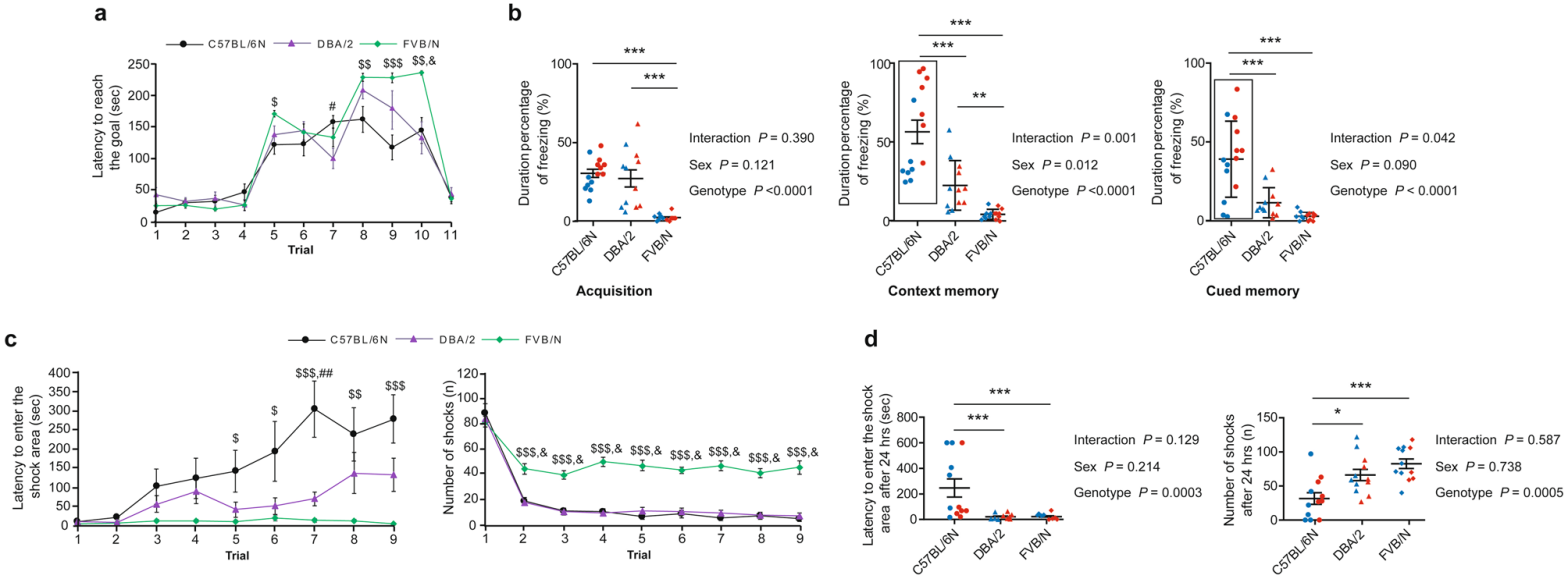
Cognitive function in C57BL/6N, DBA/2, and FVB/N mice. (**a**) In the puzzle box, FVB/N mice showed significantly higher latency to reach the goal in the first sawdust trial (trial 5) and in all trials of the plug (Trials 8, 9, and 10) compared to C57BL/6N mice. DBA/2 mice showed similar results to C57BL/6N mice in all trials except trial 7 showing a decreased latency. (**b**) In the fear conditioning test, FVB/N mice showed less duration of freezing in the acquisition and context memory trials compared to C57BL/6N and DBA/2 mice. In the cued memory trial, C57BL/6N mice showed a significant increase in the duration of freezing compared to DBA/2 and FVB/N mice. (**c**) In the active place avoidance test, FVB/N mice showed an increased number of electrical shocks compared to C57BL/6N and DBA/2 mice in all trials except the pre-training trial 1. FVB/N mice showed a significantly decreased latency to enter the shock area compared to C57BL/6N mice in trials 5–9. (**d**) C57BL/6N mice showed a significantly decreased number of theoretical electrical shocks and increased latency to enter the shock area compared to DBA/2 and FVB/N mice after 24 h. Two-way ANOVA followed by Tukey post hoc test, ($*P* ≤ 0.05, $$*P* ≤ 0.01, $$$*P* ≤ 0.001 FVB/N vs. C57BL/6N); (#*P* ≤ 0.05, ##*P* ≤ 0.01 DBA/2 vs. C57BL/6N); (&*P* ≤ 0.001 FVB/N vs. DBA/2). For (**b**) and (**d**), *P ≤ 0.05, **P ≤ 0.01, ***P ≤ 0.001. A black rectangle indicates a significant difference between sexes within a strain (refer to Supplementary Table 1). Blue and red dots refer to males and females, respectively. Error bars indicate the standard error of the mean (SEM).

The puzzle box test evaluates the ability of a mouse to solve simple (trials 1 and 11) and increasingly complex tasks (trials 2 and 3: underpath; 5 and 6: sawdust filled underpath; 8 and 9: cardboard blocked underpath) to escape an unpleasant surrounding and measures memory retrieval by one test trial 24 h after two learning trials (trials 4, 7, and 10). Young C57BL/6N mice mastered the increasing difficulties with an expected trend towards a higher latency in the memory performance after 24 h (Fig. 4a). Interestingly, DBA/2 mice showed a very good ability to memorize how to perform the trial successfully with lower latency to enter the target zone in trials (4, 7, and 10) and reached significance versus C57BL/6N mice in trial 7 (removing the sawdust) (*P* = 0.0118) (Fig. 4a). Generally, FVB/N mice exhibited the highest latency to reach the goal and the lowest ability to learn the difficult trials of removing the cardboard plug (8, 9, and 10) (trial 8: *P* = 0.001 vs. C57BL/6N and 0.596 vs. DBA/2; trial 9: *P* = 0.0003 vs. C57BL/6N and 0.139 vs. DBA/2; trial 10: *P* = 0.005 vs. C57BL/6N and 0.001 vs. DBA/2) (Fig. 4a).

#### Fear conditioning

In a classical fear conditioning test, the mouse was confronted with a conditioned and unconditioned stimulus, where it could not escape the unpleasant unconditioned stimulus. Analyzing cued and contextual fear memory in young C57BL/6N, DBA/2, and FVB/N mice revealed that for acquisition, context and cued memory, FVB/N mice did not learn to avoid an electrical shocks trial (Fig. 4b). The C57BL/6N strain showed a better pain memory than the DBA/2 strain in the context as well as the cued memory as shown by the higher duration percentage of freezing (context memory: *P* < 0.0001 and cued memory: *P* < 0.0001) (Fig. 4b). Interestingly, and independent of the high fear conditioning, females C57BL/6N mice showed an additional significant increase in the duration percentage of freezing compared to male mice (context memory: *P* < 0.0001 and cued memory: *P* = 0.009) (Fig. 4b; Supplementary Table 1).

#### Active place avoidance

In the active place avoidance test, spatial learning and memory, a hippocampus-dependent task, is measured on successive trials on the same day and after 24 h*.* One randomly chosen 60° sector on a rotating platform was designated as a non-rotating shock zone. Entering this sector, the mouse received an electric shock, which was repeated every 2 s when the mouse failed to leave the sector. Although the three strains showed similar behavior in the pre-training trial 1 without the foot shocks, FVB/N, distinct to C57BL/6N and DBA/2 mice were unable to learn to avoid the shock zone. This accounted for the decreased latency to enter the shock area and the increased number of shocks (Fig. 4c). Testing these mice after 24 h unraveled that the C57BL/6N strain showed a higher level of memory as shown by the increased latency to enter the shock area (*P* = 0.001 vs. DBA/2 and FVB/N) and the decreased number of theoretical shocks (*P* = 0.015 vs. DBA/2 and 0.0004 vs. FVB/N) (Fig. 4d). No difference between male and female mice was seen in all three strains (Supplementary Table 1).

## Discussion

Studying the behavior of rodents contributes to the understanding of the pathophysiology of neuropsychiatric disorders and paves the way for new therapeutics. Particularly, investigating behavior during the developmental time window that matches the onset of symptoms in humans before or around puberty corresponding to P42 in mice^21,37^ may improve the validity of mouse models. However, to our knowledge, there is no behavioral study in young mice thoroughly exploring the main behavioral parameters measuring the brain functions known to show deficits in neuropsychiatric disorders. Filling this important gap, we used a portfolio of diverse behavioral paradigms covering the classical behavioral testings as well as voluntary and observer-independent behavioral measurements related to neuropsychiatric phenotypes. We investigated adolescent mice of three wild-type strains frequently used as mouse models of neuropsychiatric disorders and took into account potential sex differences. We will discuss that these behavioral analyses of adolescent mice likely allow more accurate phenotyping of neuropsychiatric disorders and facilitate controlling drug effects.

Our behavioral test battery covered the main domains of altered behaviors in neuropsychiatric disorders including hyperactivity, anxiety, sensory manifestations, social deficits, and cognitive dysfunction^18^, the scope of several behavioral tests being not limited to one domain. We found that adolescent mice from different genetic backgrounds exhibited distinct behavioral patterns related to sociability, memory function, and even innate behaviors. Thus, the genetic background is, in part, responsible for strain-specific behavioral phenotypes and potential predisposition to some neurological disorders.

The automated LABORAS monitors a wide range of innate behaviors under highly standardized conditions over a prolonged period (24 h) independent of an experimenter. This is important as young mice are more hectic and more prone to the impact of the environmental milieu. To cope with this constraint, the LABORAS experiment was done at P36 and P46, at least 15 days after weaning, where the effect of isolation is suggested to be minimal. In short, at P36, the high activity of FVB/N mice indicated by increased locomotion and average speed is consistent with previous studies in adult mice^27^. Repetitive behaviors are associated with ASD^38^ and other neuropsychiatric disorders including ADHD and obsessive–compulsive disorder^39^. The increased repetitive rearing and climbing behavior in FVB/N mice may mask the repetitive behavioral endophenotype of these disorders in gene-modified FVB/N mouse models. Thus, care should be taken choosing this mouse strain for testing exploratory and repetitive behaviors.

Repeating the LABORAS measurements at P46 with the inclusion of DBA/2 mice confirmed the hyperactivity of FVB/N mice and unraveled the low activity of DBA/2 mice. Notably, several significant differences were observed between P36 and P46 mice, indicating the high capacity during adolescence towards developmental progress. Thus, several activities—distance, average speed, eating—were increased in both C57BL/6N and FVB/N strains at P46. But, while the maximum speed of FVB/N mice was increased, that of C57BL/6N mice was decreased at P46 compared to P36. In FVB/N mice, repetitive rearing and climbing increased, whereas self-grooming decreased from P36 to P46. As a few days difference can have a strong impact on the behavioral pattern, these results emphasize the importance of comparing mice of the same age.

Nest building is an important indicator of health and welfare in adult laboratory mice. Moreover, it is an indicator of sociability^40,41^ and can be affected by aggression within the cage, thermal stress and pain^42^. P30 and P40 mice showed a poor nest-building capacity, with the ability of FVB/N exceeding that of C57BL/6N and DBA/2. These results are in contrast with a study by Moy et al.^41^ demonstrating C57BL/6J, DBA/2J, and FVB/NJ mice building nests already at 3–4 weeks of age. Distinct to our study, nest building was performed at 10 a.m. and investigated at 7 p.m. with 3–4 mice in the same cage. Comparing the two studies emphasizes an unexpectedly high impact of the time of experiments and housing conditions on nest-building.

Burrowing is a sensitive method for detecting behavioral dysfunction^33^. It is important in assessing hippocampal function and integrity^43^ and is impaired in mice with hippocampal lesions^44^. Adolescent mice of the three strains were able to burrow the food pellets from the tube. Only during the overnight stage, FVB/N mice burrowed more food pellets than C57BL/6N and DBA/2.

The open field, dark/light compartment, and elevated plus maze tests are widely used measurements of anxiety-like behavior in mice and for assessing the efficacy of anxiolytic drugs. Strain differences in murine anxiety paradigms are well established in adult mice^30,45–47^, and include mouse mutants as well as pharmacological studies^48,49^. Anxiety tests are based on the natural aversion by mice to open, elevated, or brightly lit spaces, where applying different behavioral experiments is recommended to cover different types of anxiety-related behavior^50,51^. C57BL/6N, DBA/2, and FVB/N mice showed similar total distance in a new arena within 10 min. This may indicate that the hyperactivity of FVB/N mice in the LABORAS cages is restricted only to a home-cage-like environment. The latency, numbers of visits, and duration in the center of the arena were also comparable in the three strains. These findings differ from a study of 5–6 weeks old mice, which revealed increased total distance and time in the center of the arena of FVB/NJ compared to C57BL/6J and DBA/2J mice^52^. Of note, the test was performed for only 5 min. Nonetheless, adult FVB/N mice also showed higher activity than C57BL/6N mice in the open field test (increased duration in the center of the arena)^53^. Interestingly, DBA/2 mice showed higher anxiety levels in the dark/light compartment and elevated plus maze tests, whereas 8–10 weeks old C57BL/6J showed more entries and duration in the open arms in elevated plus maze test than DBA/2^52^, which is consistent with our results in adolescent mice. Moreover, similarity between the C57BL/6N and FVB/N strains is consistent with a previous finding in adult mice^27^. Adult FVB/NJ mice also showed an increased percentage of duration in the open arms compared to DBA/2 mice^54^. The low anxiety-like behavior levels of FVB/N mice in the dark/light compartment and elevated plus maze tests was confirmed in another experimental apparatus. FVB/N showed more head pokes in the hole-board test compared to both C57BL/6N and DBA/2, indicating more activity and less anxiety to explore the new environment. This highly exploratory behavior of FVB/N mice in the hole-board test is also consistent with the high activity in the LABORAS cages.

Taken together, strain differences in anxiety were repeatedly reported^55–57^ and adult DBA/2J mice were frequently characterized by anxiety-related responses and high or intermediate-high emotional reactivity^47,58^. The high anxiety of DBA/2 mice may mask the anxious behavior in genetically-modified mouse models and should be taken into consideration in designing the experiments and analyzing the data. Importantly, anxiety-related behavioral experiments of adolescent and adult mice^27,52,59,60^ do not always reflect anxiety levels. As different anxiety paradigms apparently tax distinct aspects of anxiety, suggesting that a battery of different tests should be used in studies of anxiety-related behaviors^59^.

Individuals with several neuropsychiatric disorders including SZ, ASD, and ADHD often display sensory manifestations secondary to the core features of the disorders accompanied by sensory processing deficits^61–67^. Therefore, we investigated whether behavioral tests measuring sensory inputs and nociception can be applied to mice at a young age. The cold plate test is a standard procedure evaluating the responses of unrestrained mice to low-temperature stimulation of the plantar aspect of the paw^68^. Adolescent C57BL/6N, DBA/2, and FVB/N mice responded similarly to the cold temperature and most of the mice managed bearing the cold temperature. None of the mice showed cold hyperalgesia, and the latency response to 2 °C is mostly in the range of 20 to 30 s that is comparable to adult mice^69,70^. Thus, the cold plate assay is suited for adolescent mice.

Social interactions are frequently distorted in patients with neuropsychiatric disorders. The three mouse strains interacted with their littermates in the direct social interaction test. C57BL/6N mice showed the highest social ability indicated by the decreased latency and increased counts of proximity. Social behavior was previously evaluated in juvenile (5–6 weeks) C57BL/6J, DBA/2J, and FVB/NJ male mice in the three-chamber social test^41^. Different to the direct social interaction, the three-chamber social test includes limitations of social interaction by the presence of a wire mesh cylinder barrier and a bigger arena area, which curtails the option to socialize directly^24^. Irrespective of these differences, the authors reported a similar preference spending time in the chamber containing a strange mouse than in exploring the empty chamber^41^. Although the strains showed similar results regarding the duration spent in the chambers, DBA/2J entered less frequently than C57BL/6J and FVB/NJ. In another three-chamber social test using 6–7 weeks old mice, C57BL/6J, DBA/2J, and FVB/NJ again showed similar duration in the chamber, but less entries of DBA/2 than C57BL/6J and FVB/NJ^52^. However, the same group reported that by using altered housing conditions, DBA/2J completely failed to show a significant sociability^54^.

In brief, all these studies including ours are confirming that the duration of contact and number of visits does not essentially correlate. One might speculate that the number of visits is affected by the degree of anxiety, whereas the duration more directly reflects social interaction. In concern about our question on the age importance, social behavior does not appear to be age-related. Finally, for approving the impact of genetic manipulation, C57BL/6N mice may be the preferred choice, whereas for drug efficacy evaluation, less social DBA/2 or FVB/N mice could be advantageous.

A combination of three or more learning and memory tasks with diverse sensory and motor demands is mandatory to strengthen the findings of fundamental cognitive abnormalities in mutant lines^17^. The memory deficits in the puzzle box are suggested to be related to hippocampal dysfunction as shown in hippocampus-lesioned mice^71^, while the memory function in the active place avoidance test is based on a cross-talk between the hippocampus-dependent contextual memory and amygdala-dependent emotional memory^72^. In the puzzle box test, C57BL/6N mice revealed the best ability to reach the goal with lower latency than both DBA/2 and FVB/N strains in most of the trials especially the most difficult ones with the cardboard plug. These results were recapitulated in two other tests for measuring learning and memory including the active place avoidance and fear conditioning tests. Interestingly, DBA/2 mice showed similar results to C57BL/6N in the active place avoidance test during the first day indicating the ability to learn the task, though at a moderate level. In contrast, FVB/N mice were absolutely incapable managing both spatial learning and the 24 h memory task. One possible explanation for the inability of the FVB/N to master these tests is their hyperactivity that can result in a lack of attention and might overshadow the ability to learn. Different to active place avoidance, DBA/2 mice showed better long-term memory than C57BL/6N mice in the puzzle box, suggesting different brain regions contributing to memory building in those two assays.

In contrast to FVB/N, both C57BL/6N and DBA/2 mice learned the fear conditioning test during the acquisition phase, where C57BL/6N mice showed the best ability for context and cued memories. These results are consistent with studies in adult mice revealing a better context memory of C57BL/6N than DBA/2 mice^55,73,74^ and better context and cued memory of C57BL/6N than FVB/N mice^53^. Difference in the three strains regarding some learning and memory tasks may rely on functional differences in the hippocampus formation^73^. Adult FVB/N mice showed deficits in the Morris water maze test^27,75,76^ and other non-visual tasks such as fear conditioning^77,78^. The FVB/N strain carries the rd mutation^79^ for retinal degeneration that affects their behavior in cognitive tests. However, as FVB/N mice also show cognitive impairments in tasks not needing vision, it is suggested that they have an initial cognitive impairment that becomes accentuated in assays relying on visual stimuli such as the Morris water maze^76^. Cognitive tasks represent endpoints that also may be affected by anxiety-related conditions. However, our findings strengthen the low cognitive ability of FVB/N mice not relying on anxiety as this strain showed low anxiety levels in the dark/light compartment and elevated plus maze tests.

Taken together, young FVB/N mice are not suitable as background strains for transgenic models that aim elaborating a decline in cognitive ability. DBA/2 mice are, as well, not suited as they show a high-frequency hearing loss as early as 3 weeks of age caused by Cdh23753G > A^80^ and Fscn2326G > A alleles^81^, which could partly explain the severe cued memory deficits compared to less severe contextual memory deficits in the fear conditioning test. Thus, the striking strain-dependent differences in the sensory development need attention when choosing behavioral tests to pinpoint neuropathological alterations.

Several sex-related differences have been described in adult mice^82–87^, but behavioral experiments on female mice are underrepresented based on the assumption that females are intrinsically more variable than males due to the estrous cycle^88^. However, this belief has been questioned by a meta-analysis reporting on comparable variability in male and female mice in a wide range of assays^89^. In view of this still controversially discussed matter, our behavioral studies in adolescent mice became of particular relevance as the susceptibility to neuropsychiatric disorders differs between males and females with a ratio of 4:1 in ASD^38,90–92^, 1.4:1 in schizophrenia^93,94^, and 1:2 in depression^95–101^. We saw a difference in behavioral experiments between adolescent male and female C57BL/6N and FVB/N but not DBA/2 mice. Interestingly, no difference in social interaction was shown between males and females consistent with a similar count of USV that was previously shown at the same age during the direct social interaction test^24^. Female C57BL/6N mice were more active and less anxious in the open field with better context and cued memories. On the other hand, female FVB/N mice exceeded males in sociability-related nesting. We conclude that sex differences during adolescence are likely restricted to certain strains and tasks as shown for adult mice^102^. Nonetheless, in view of the divergent susceptibility of male and female to neuropsychiatric phenotypes, there is an urgent need for tackling this question.

Finally, we want to touch a general problem in behavioral studies. The behavioral neuroscience field suffers from the issue of reproducibility of behavioral results in genetically-modified mice such that it was suggested that behavioral analyses may be too unstable for capturing fine-scaled genetic differences^103^. Potential confound factors include species and environmental variability such as breeding and housing conditions along with different handling and disparity in the used apparatuses^104–106^. As high standardization may facilitate reproducibility^107^, many automated methods are now replacing manual ones such as LABORAS monitoring home-cage-like behaviors and automated cages for measuring learning abilities. Moreover, although individuals of an inbred strain are meant to be genetically identical, they may still differ in minisatellite regions, short repetitive DNA sequences with highly polymorphic copy numbers, which potentially affect gene expression and behavior^108^. Other uncontrollable environmental influences, such as the intrauterine position of the embryo and feeding hierarchy in newborns can cause within-strain variability as shown in some results of our study and have to be accepted as part of research variables^108^, which only can be coped with by large numbers and/or repetitions, preferably in independent laboratories. Notably, across a multidimensional set of 115 behavioral parameters, several strains consistently ranked high in within-strain variability (DBA/2J, 129S1/Sv A/J and NOD/LtJ), whereas other strains ranked low (C57BL/6J and BALB/c)^109^. We additionally recommend having consistently alike male/female ratios as according to our findings, differing ratios can shift the results. Taking the effects of small developmental progress, it is also important to only incorporate control littermates of the same delivery day as knockout/knockin mice. Two additional aspects require consideration measuring behavior of young mice. First, young mice are very sensitive to isolation from the littermate. Therefore, the paradigms that need single housing (home-cage-like behaviors, nesting test, and burrowing) should be conducted at a later stage of the development to reduce social bias. Second, young mice are more active and hectic than adult mice, and habituation session like handling of the animals before starting a task is practically not applicable. Hence, the animal-experimenter interaction is of greater importance than in adult mice, and handling should be performed by the same person in all testings within one study. Finally and self-understanding, for the statistical analysis, the expected variability in young mice has to be taken into account and requires higher numbers. As mentioned, different behavioral experiments measuring the same endophenotype, e.g. anxiety, can show assay-dependent divergent results. Thus, we propose performing the whole behavioral ethogram panel.

Summarizing published and our results on behavioral studies in mouse models of neuropsychiatric disorder, design and result interpretation requires special attentions. As a rule, several behavioral test batteries should be used. It is unlikely that all symptoms of a neuropsychiatric disorder has parallels in a single strain or knockout mouse. Instead, within the available armamentarium of mouse models, at least one may offer the desired selective phenotype^52,110^. In addition, many neuropsychiatric disorders share comorbid symptoms and therefore, the choice of the strain according to the expected endophenotype is mandatory. Accordingly, we do not recommend FVB/N strain for testing cognitive and locomotor functions, and the high anxiety levels of DBA/2 mice may mask the anxiety-like phenotype of neuropsychiatric disorders. Instead, adolescent C57BL/6N mice displayed well in our behavioral test battery, except for nesting. The proper choice of tests and mouse models being an utmost important matter should be based on profound knowledge of behavioral genetics and the specific goals of the study.

In brief, we suggest adolescent rather than adult mice being suited for behavioral experiments related to neuropsychiatric disorders as patients frequently display first symptoms during adolescence. We also stress the importance of small developmental windows, which helps decreasing variability and concomitantly allows defining “disease progression”. First evidences for sex-related differences in adolescence helps in deciding on models for neuropsychiatric disorders preferentially observed in men and women. Our behavioral studies in adolescent mice can also be used as a guiding platform for testing drug efficacy in different behavioral abnormalities.

## Material and methods

### Mice and housing conditions

Breeding pairs from male and female C57BL/6N, DBA/2, and FVB/N mice were purchased from Charles River Laboratories (Sulzfeld, Germany) at the age of 8 weeks. Mice for the behavioral tests were expanded at the Interdisciplinary Neurobehavioral Core, University of Heidelberg. Mice were housed in groups of three per cage. All mice were housed with food and water *ad libitum* under a standard 12-h light/dark cycle (7:00 p.m.–7:00 a.m.) with a regulated ambient temperature of 22 °C and at a relative humidity of 40–50%. All procedures were conducted in strict compliance with national and international guidelines for the Care and Use of Laboratory Animals. Extreme care was taken to minimize suffering for the animals. Animal experiments were approved by the local governing body (Regierungspräsidium Karlsruhe, Germany G-100/16; G-103/16; G-105/16).

### Experimental design and groups

Pregnant females were isolated, and pups were weaned on a postnatal day 21. All behavioral tests were performed after weaning and were carried out during the daylight cycle except for nesting and burrowing tests which were performed overnight. Mice were brought into the behavioral room 30 min before test beginning without prior handling. Experiments started at 7 a.m. except for nesting and burrowing tests which started at 5 p.m. and hole-board test that was performed at 2 p.m. The behavioral experiments were performed on male and female mice from 10 different cohorts to decrease the stress on these young mice and avoid the effect of prior experiences. The number of mice per each cohort and the type of the behavioral experiment are listed (Table 1). After each trial, the apparatuses were carefully cleaned with 75% ethanol solution wetted tissue paper. All behavioral experiments were performed in a randomized manner with a separation of experiment conduction and data analysis.

**Table 1:** Mouse cohorts and age of the adolescent mice used in the different experiments.

Cohort	Strain	Mice (#)		Behavioral test at postnatal day (P#)
		Male ♂	Female♀	
1	C57BL/6N	6	7	Open field (P22)
	DBA/2	5	8	Dark/light compartment (P25)
	FVB/N	7	8	LABORAS; C57BL/6N and FVB/N (P36)
2	C57BL/6N	7	7	Hole-board (P22)
	DBA/2	12	6	
	FVB/N	8	8	
3	C57BL/6N	8	6	Puzzle box (P23–P26)
	DBA/2	10	6	
	FVB/N	7	8	
4	C57BL/6N	13	10	Direct social interaction (P24–P28)
	DBA/2	6	6	
	FVB/N	7	11	
5	C57BL/6N	7	8	Nesting (P30)
	DBA/2	9	6	Burrowing (P31)
	FVB/N	9	8	Nesting (P40)
6	C57BL/6N	7	8	Cold plate (P32)
	DBA/2	4	5	
	FVB/N	9	8	
7	C57BL/6N	6	6	Active place avoidance (P33–P35)
	DBA/2	6	6	
	FVB/N	7	5	
8	C57BL/6N	7	7	Fear conditioning (P37–P40)
	DBA/2	6	6	
	FVB/N	7	5	
9	C57BL/6N	6	6	Elevated plus maze (P38)
	DBA/2	5	8	
	FVB/N	7	8	
10	C57BL/6N	8	7	LABORAS (P46)
	DBA/2	8	9	
	FVB/N	9	8	

### Behavioral test battery

#### Home cage monitoring (LABORAS)

The LABORAS (Laboratory Animal Behavior Observation Registration and Analysis System; Metris B.V.) is an advanced and non-invasive system that uses a carbon fiber platform to detect behavior-specific vibration patterns produced by the animal^111,112^. Each mouse was tested individually for 24 h in a cage placed on top of the platform under standard housing conditions with free access to food and water. The specific LABORAS software version 2.6 processed the produced vibrations into various behavioral parameters including the duration and frequency of locomotion, climbing, rearing, self-grooming, drinking, and eating. The LABORAS experiments were performed at P36 for C57BL/6N and FVB/N and P46 for C57BL/6N, DBA/2, and FVB/N.

#### Nesting test

A mouse was put at 5 p.m. in a new home cage with a cotton nesting material (Nestlet, Datesand, Stockport) and fresh bedding and checked the next morning at 7 a.m. for nest-building. The nest quality was assessed with a complexity score from 1 (no nest) to 5 (complex nest with a wall surrounding the mouse)^113,114^. The nesting test was performed at P30 and P40.

#### Burrowing test

The burrowing test is based on the mouse behavior to displace items from a tube within their home cage^33,114^. The tube was filled with 200 g of food pellets covered on top with 60 g of fresh bedding. The test was performed at 5 p.m. for each mouse individually, and the remaining pellets and bedding in the tube was weighted after 2 h and then returned into the tube. After 16 h, the remaining pellets and bedding in the tube was weighted again. The burrowing test was performed at P31.

#### Open field test

The baseline activity was measured by placing each mouse individually in the center of a 40 × 40 cm^2^ white box with 40 cm high walls for 10 min. The light intensity was 290 lx in the center of the arena. The mouse activity was digitally recorded using a video camera placed 1 m above the center of the arena. The automatic detection of the mouse path was analyzed with the SYGNIS tracker software (SYGNIS). Besides the analysis of the general locomotion, the latency, duration, and the number of visits by the mouse to the inner arena (10 × 10 cm^2^) away from the wall were calculated for measuring the anxiety level. The open field test was performed at P22.

#### Dark/light compartment test

The dark/light compartment test has been established to study anxiolytic drug effects and is widely used to assess the level of anxiety in rodents^115–117^. The dark–light box is an open white rectangle (40 × 40 × 33 cm^3^) attached to an 8 × 10 cm opening to a dark compartment (with a lid and painted in black) (20 × 40 × 33 cm). In our study, the light compartment was illuminated with 500 lx. Each mouse was put in the dark chamber and the latency, as well as the number of visits to the light compartment within 10 min, were measured. Only when all four limbs of the subject crossed the entrance, it was considered as an entry to the light compartment. The behavior was video recorded with a digital camera and tracked using the SYGNIS tracker software (SYGNIS). The dark/light compartment test was performed at P25.

#### Elevated plus maze test

The elevated plus maze test assesses the anxiety-like behavior by measuring the conflict between the natural tendency of mice to explore a novel environment and the aversive properties of an open arena^118,119^. The maze is a crossed-shaped platform (grey opaque plastic material) with equally sized arms (8 × 30 cm^2^) and a central intersection (8 × 8 cm^2^), allowing mice to move freely into each zone of the maze. Two of the arms (opposing each other) were flanked by 17 cm high opaque walls (closed arms); the remaining two arms are without walls (open arms). The maze is elevated 70 cm above the floor. The central intersection, open, and closed arms were illuminated by 230, 230 and 160 lx, respectively. Each mouse was placed in the central intersection facing one of the closed arms and allowed to explore the maze freely for 10 min. The behavior was monitored with a digital camera and tracked with the SYGNIS tracker software (SYGNIS). The ratios of the sum of visits and duration in the two open arms compared to the closed arms were calculated. The elevated plus maze test was performed at P38.

#### Hole-board test

The hole-board test takes advantage of the natural tendency of mice to dip their head into holes. It is mainly used to study the behavior of the mouse confronted with a new environment and could thereby give insights into general and anxiety-like behaviors^34–36^. The automated hole-board system (Ugo Basile) is made of grey Perspex material with a dimension of 40 × 40 cm^2^, 2.2 cm thick with 16 holes of 3 cm diameter, spaced 10 cm apart. Infrared photocells in the holes automatically counted the nose-poking or head-plugins during 10 min test duration. The hole-board test was performed at P22.

#### Cold plate test

The cold plate test was performed with a Hot/Cold Plate (Bioseb, Vitrolles) at 2 °C for the assessment of the analgesic response^68,120^. The latency until the first withdrawal response of the hind paw was recorded, and the mouse was removed immediately. Cut-off latencies were set at 30 s. The test was repeated three times with 5 min intervals. The average of the three trials was calculated. The cold plate test was performed at P32.

### Social interaction

For measuring the direct social interaction, one mouse was isolated from the littermate for 24 h in a separate colony housing room. The social test included 2 trials with trial 1 serving as a habituation phase by placing one mouse from the colony into a white acryl open field box (40 × 40 × 40 cm^3^) for 2 min. In trial 2, the same-sex sibling mouse was placed gently next to the isolated mouse for 5 min. The social interaction was videotaped and analyzed using the EthoVision XT software (Noldus). The proximity was considered when the difference between the centers of the two tested mice is equal to or less than 10 cm. The centers of the mice were recognized by the software as part of the mice's backs were shaved the previous day and dyed with different colors using animal marking stick (MS Schippers, AH Bladel). The direct social interaction test was performed between P24 and P28.

### Puzzle box test

The puzzle box test was slightly modified from the one described in^71^ and performed between P23 and P26. The home-made puzzle box consisted of two compartments (a brightly-lit start compartment—illuminated with a bright light (320 lx) and a smaller closed, dark compartment made of grey and plastic material, respectively. Both compartments were separated by a black plastic wall that had a narrow door (about 4 cm wide) with an underpass (depth 2 cm). In each trial, the mouse was placed in the start zone, and the task was to enter the goal zone which contained bedding from its home cage. Each mouse underwent a total of 11 trials over 4 consecutive days, with 3 trials per day on day 1–3 and 2 trials on day 4. During trial 1, the door and the underpass were open and the mouse could use the open door to escape from the aversive illuminated light compartment into the home zone. In trials 2 and 3, the door was blocked and the mouse had to use the small underpass to enter the goal zone. Trial 4 was identical to trials 2 and 3. In trials 5 and 6, however, the underpass was filled with sawdust and the mouse had to dig through the sawdust to reach the home zone. Trial 7 was identical to trials 5 and 6. During trials 8 and 9, the mouse had to deal with an underpass that was blocked by a cardboard plug. The mouse had to remove the plug using its teeth and/or paws to enter the goal zone through the underpass. Trial 10 was a repetition of trial 9. In the last trial, trial 11, trial 1 was repeated as a control trial. After trial 1–10, each mouse was left for 1 min inside the goal zone even if the mouse could not reach the home zone on its own.

### Fear conditioning test

The fear conditioning test evaluates natural fear learning as it has been described before^121^. Mice spent 6 min inside a conditioning chamber (Med Associates Inc., St. Albans, Vermont) and were exposed to 4 acoustic signals (5 kHz, 85 dB, 30 s each) at 90–120 s, 150–180 s, 210–240 s, and 270–300 s (conditioned stimulus, CS). At the last second of each tone segment, a foot shock (0.6 mA, 1 s) was applied *via* the floor grid (unconditioned stimulus, US). Freezing behavior was analyzed via a video camera connected to a video tracking software (Med Associates Inc.) to enable measuring the freezing numbers and durations using “Video Freeze” software. After 24 h, animals were re-introduced to the chamber for 6 min in the absence of CS or foot shocks to evaluate the contextual fear memory. 48 h after training, the cued fear memory was assessed. To hinder the recognition of the chamber from haptic, olfactory, or visual cues, the chamber was remodeled with a flat plastic floor panel covering the steel grid and a black roof-shaped insert, by replacement of visible light with near-infrared illumination, and by use of a different disinfectant for cleaning. Mice were exposed to this altered context for 4 min during which the 30 s CS was presented twice, terminating at 60 and 180 s, respectively. Freezing behavior was analyzed as described before. The fear conditioning test was performed between P37 and P40.

### Active place avoidance test

The active place avoidance paradigm, which is sensitive to hippocampal dysfunction, was used to assess spatial reference memory as it has been described before^121^. The active place avoidance apparatus was located in a testing cubicle providing visual cues. It consisted of a rotating circular platform surrounded by a transparent wall. One randomly chosen 60° sector was designated as the non-rotating shock zone that was stationary to the spatial signals in the test room. When a mouse entered this 60° sector, the mouse received a 0.4 mA electric shock upon entry, and further identical shock every 2 s after failure to leave the sector. For mice, this sector was only identifiable relative to the extra-maze visual cues. Due to the platform rotation, passive strategies were inevitably associated with foot-shocks, and mice had to learn actively to avoide the sector. After one 10 min pre-training trial without electric shocks, eight 10 min training trials were conducted with a 15 min inter-trial interval during which the mice were placed in their home cages. After 24 h, one 10 min retention trial without shocks was performed. The latency to enter the shock area and the number of potential shocks were recorded during all trials. The behavior was monitored with a digital camera and tracked with the SYGNIS tracker software (SYGNIS). The active place avoidance test was performed between P33 and P35.

### Statistical analysis

Two-way ANOVA was used with sex and genotype as the two factors. This was followed by Tukey’s post hoc test for multiple comparisons to determine differences between the three strains C57BL/6N, DBA/2, and FVB/N and Bonferroni correction to check differences between males and females within each strain. To compare the LABORAS results between P36 and P46 for C57BL/6N and FVB/N, two-way ANOVA was used with sex and age as the two factors. All data were expressed as mean ± SEM. A *P*-value ≤ 0.05 was considered statistically significant. Statistical analysis was performed using GraphPad Prism 7 and Microsoft Office Excel software. The respective numbers of male and female mice are described in Table 1 and the individual figures.

## Supplementary information

Supplementary information
