# Supplementary material for: Behavioral tests assessing neuropsychiatric phenotypes in adolescent mice reveal strain- and sex-specific effects
Source: Sci Rep. 2020 Jul 9;10:11263. doi: 10.1038/s41598-020-67758-0 (PMC7347854; doi:10.1038/s41598-020-67758-0)
Supplement: Supplementary file 1 — Supplementary information [file 41598_2020_67758_MOESM1_ESM.docx]

**Behavioral tests assessing neuropsychiatric phenotypes in adolescent mice reveal strain- and sex-specific effects**

**Ahmed Eltokhi^1,2^*, Barbara Kurpiers^2^, Claudia Pitzer^2^***

^1^ Department of Neurology and Epileptology, Hertie Institute for Clinical Brain Research, University of Tübingen, Tübingen, Germany

^2^ Interdisciplinary Neurobehavioral Core, Heidelberg University, Heidelberg, Germany


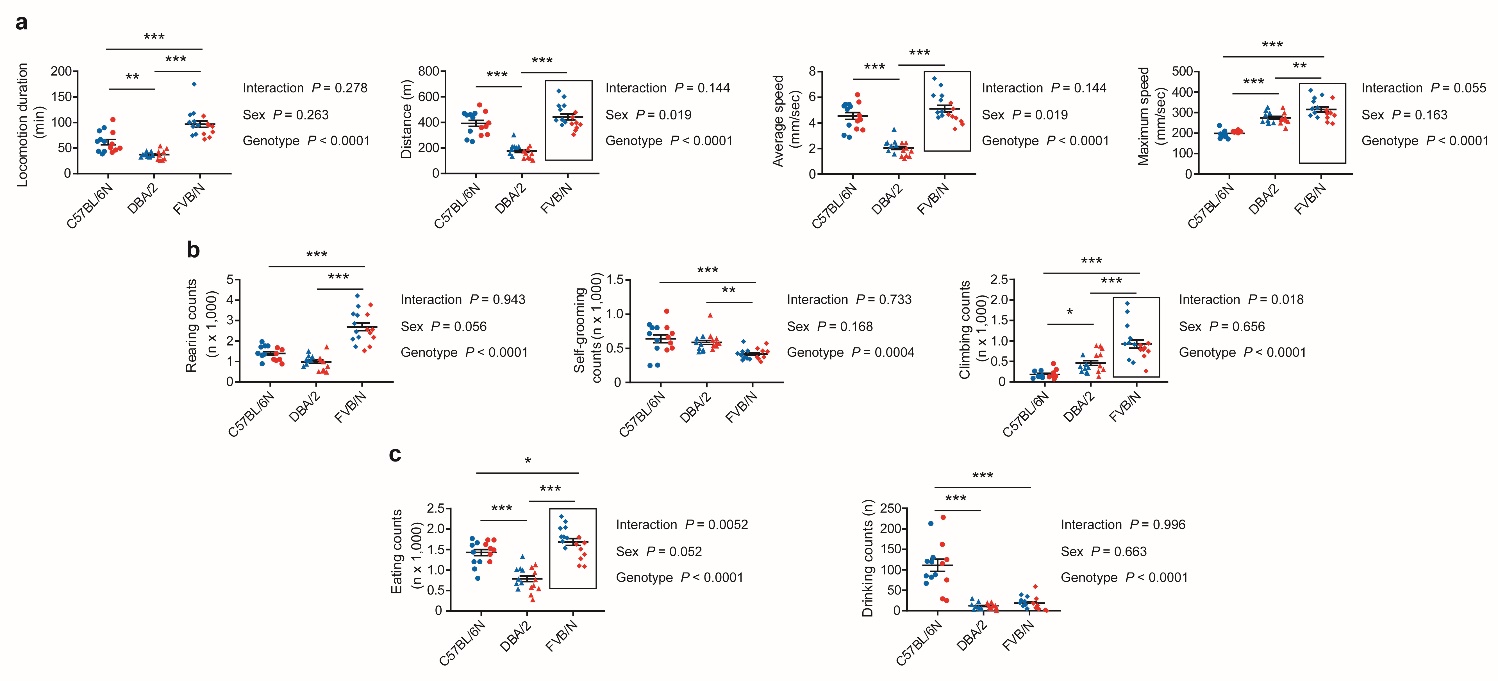


**Supplementary Figure 1:** The **LABORAS test for C57BL/6N, DBA/2, and FVB/N mice at P46 in which mouse movements were continuously monitored for 24 hrs.** a) The FVB/N strain showed the highest activity, the DBA/2 strain exhibited the lowest activity and C57BL/6N strain was intermediate between the two strains. b) Increased repetitive rearing and climbing behaviors and decreased self-grooming in FVB/N compared to C57BL/6N and DBA/2 mice. c) DBA/2 mice showed decreased eating counts compared to C57BL/6N and FVB/N mice. C57BL/6N mice showed increased drinking counts compared to DBA/2 and FVB/N. Two-way ANOVA followed by Tukey post hoc test,**p* ≤ 0.05, ***p* ≤ 0.01, ****p* ≤ 0.001. A black rectangle indicates a significant difference between sexes within a strain (refer to Supplementary Table 2). Blue and red dots refer to males and females, respectively. Error bars indicate the standard error of the mean (SEM).

**
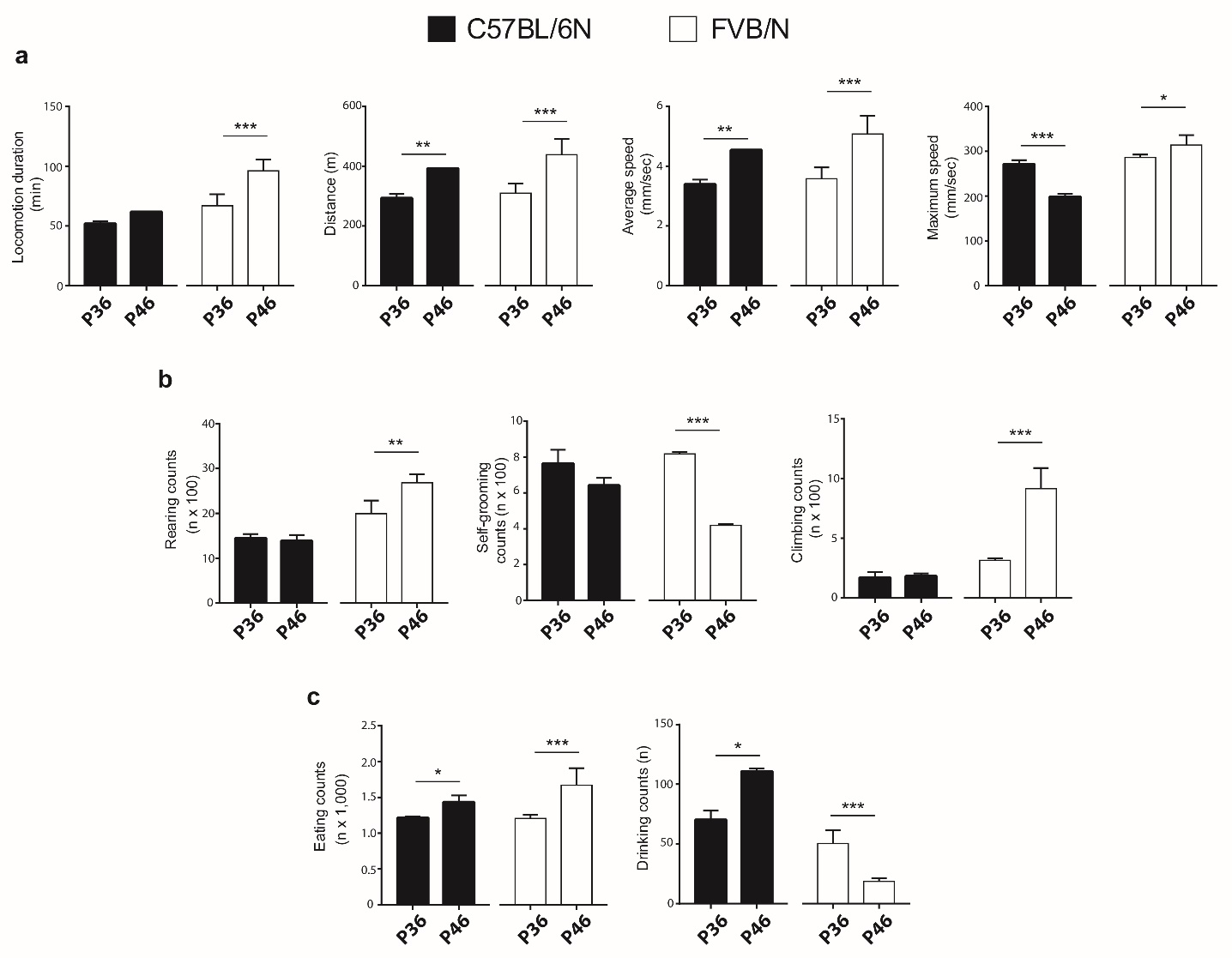
**

**Supplementary Figure 2:** **The LABORAS result comparison between P36 and P46 of C57BL/6N and FVB/N mice.** Two-way ANOVA followed by Tukey post hoc test, **p* ≤ 0.05, ***p* ≤ 0.01, ****p* ≤ 0.001. Error bars indicate the standard error of the mean (SEM).

**Supplementary Table 1: Comparison between male and female mice of C57BL/6N, DBA/2, and FVB/N strains in the behavioral test battery**

| **Name of Test** | **C57BL/6N** |  |  | **DBA/2** |  |  | **FVB/N** |  |  |
| --- | --- | --- | --- | --- | --- | --- | --- | --- | --- |
|  | ♂ Mean +/- SEM | ♀ Mean +/- SEM | Bonferroni test | ♂Mean +/- SEM | ♀ Mean +/- SEM | Bonferroni test | ♂Mean +/- SEM | ♀ Mean +/- SEM | Bonferroni test |
| **LABORAS** |  |  |  |  |  |  |  |  |  |
| Locomotion duration | 54.02  +/-  2.07 | 49.74  +/-  4.45 | > 0.999 | - | - | - | 57.27  +/-  7.55 | 76.60  +/-  5.57 | 0.034 |
| Distance | 306.94  +/-  12.30 | 279.85  +/-  17.33 | 0.842 | - | - | - | 277.45  +/-  21.18 | 341.81  +/-  29.53 | 0.094 |
| Average speed | 3.55  +/-  0.14 | 3.24  +/-  0.20 | 0.842 | - | - | - | 3.21  +/-  0.25 | 3.96  +/-  0.34 | 0.094 |
| Maximum speed | 262.33  +/-  6.10 | 280.57  +/-  13.05 | 0.371 | - | - | - | 280.29  +/-  8.06 | 292.50  +/-  7.17 | 0.672 |
| Rearing counts | 1537.33  +/-  122.58 | 1357.57  +/-  123.35 | 0.943 | - | - | - | 1704.29  +/-  179.72 | 2288.25  +/-  199.71 | 0.035 |
| Self-grooming counts | 688.83  +/-  55.73 | 841.14  +/-  106.88 | 0.383 | - | - | - | 828.86  +/-  77.27 | 808.50  +/-  55.37 | > 0.999 |
| Climbing counts | 216.50  +/-  21.57 | 124.57  +/-  16.27 | 0.632 | - | - | - | 296.00  +/-  95.62 | 332.13  +/-  62.11 | > 0.999 |
| Eating counts | 1198.17  +/-  49.64 | 1233.14  +/-  97.65 | > 0.999 | - | - | - | 1156.86  +/-  112.65 | 1258.88  +/-  186.48 | > 0.999 |
| Drinking counts | 62.50  +/-  4.22 | 77.86  +/-  17.15 | 0.789 | - | - | - | 61.43  +/-  15.69 | 39.25  +/-  4.45 | 0.382 |
| **Nesting test** |  |  |  |  |  |  |  |  |  |
| Score (at P28-32) | 1.00  +/-  0.00 | 1.00  +/-  0.00 | > 0.999 | 1.00  +/-  0.00 | 1.00  +/-  0.00 | > 0.999 | 1.33  +/-  0.17 | 1.63  +/-  0.18 | 0.173 |
| Score (at P38-42) | 1.00  +/-  0.00 | 1.29  +/-  0.18 | 0.260 | 1.00  +/-  0.00 | 1.00  +/-  0.00 | > 0.999 | 1.33  +/-  0.17 | 2.00  +/-  0.00 | 0.0003 |
| **Burrowing test** |  |  |  |  |  |  |  |  |  |
| Burrowing after 2 hr | 16.27  +/-  3.43 | 25.19  +/-  5.27 | 0.765 | 34.64  +/-  6.50 | 27.12  +/-  9.85 | > 0.999 | 28.71  +/-  3.03 | 27.54  +/-  2.91 | > 0.999 |
| Burrowing after 16 hr | 43.87  +/-  9.02 | 36.65  +/-  6.55 | > 0.999 | 67.74  +/-  7.27 | 43.35  +/-  10.32 | 0.373 | 84.62  +/-  9.43 | 96.81  +/-  28.02 | > 0.999 |
| **Open Field** |  |  |  |  |  |  |  |  |  |
| Total Distance | 50.88  +/-  2.81 | 107.20  +/-  22.90 | 0.004 | 62.59  +/-  4.23 | 83.20  +/-  8.40 | 0.678 | 61.22  +/-  3.52 | 55.20  +/-  5.40 | > 0.999 |
| Latency to the center of the arena | 21.28  +/-  12.14 | 18.40  +/-  5.50 | 0.357 | 67.12  +/-  26.12 | 45.70  +/-  17.00 | > 0.999 | 121.24  +/-  25.93 | 95.60  +/-  28.50 | > 0.999 |
| Number of visits to the center of the arena | 13.67  +/-  1.28 | 19.60  +/-  4.60 | 0.088 | 15.60  +/-  2.56 | 17.90  +/-  3.30 | > 0.999 | 13.29  +/-  2.77 | 16.40  +/-  2.60 | > 0.999 |
| Duration in the center of the arena | 53.27  +/-  3.12 | 72.00  +/-  26.40 | 0.031 | 6.78  +/-  1.34 | 7.70  +/-  1.4 | > 0.999 | 6.34  +/-  2.02 | 9.70  +/-  2.00 | > 0.999 |
| **Dark/light compartment** |  |  |  |  |  |  |  |  |  |
| Latency to the light compartment | 129.73  +/-  44.72 | 63.26  +/-  20.32 | 0.904 | 213.24  +/-  68.74 | 228.48  +/-  69.85 | > 0.999 | 20.30  +/-  4.26 | 37.53  +/-  11.94 | > 0.999 |
| Duration in the light compartment | 124.55  +/-  33.76 | 79.84  +/-  16.66 | 0.716 | 43.96  +/-  14.66 | 98.74  +/-  26.16 | 0.482 | 130.19  +/-  29.50 | 172.84  +/-  24.03 | 0.682 |
| **Elevated plus maze** |  |  |  |  |  |  |  |  |  |
| Visit ratio (open vs closed | 0.56  +/-  0.07 | 0.55  +/-  0.09 | > 0.999 | 0.34  +/-  0.06 | 0.27  +/-  0.05 | > 0.999 | 0.51  +/-  0.10 | 0.74  +/-  0.14 | 0.244 |
| Duration ratio (open vs closed | 0.26  +/-  0.053451 | 0.29  +/-  0.12 | > 0.999 | 0.10  +/-  0.02 | 0.09  +/-  0.03 | > 0.999 | 0.17  +/-  0.07 | 0.52  +/-  0.11 | 0.010 |
| **Hole- board test** |  |  |  |  |  |  |  |  |  |
| Number of head pokes | 56.86  +/-  4.77 | 36.29  +/-  3.15 | 0.165 | 43.92  +/-  5.10 | 60.83  +/-  9.46 | 0.269 | 92.13  +/-  4.78 | 107.57  +/-  13.03 | 0.301 |
| **Cold Plate tests** |  |  |  |  |  |  |  |  |  |
| Duration | 25.75  +/-  1.26 | 25.86  +/-  1.23 | > 0.999 | 29.58  +/-  0.43 | 24.16  +/-  2.79 | 0.424 | 25.43  +/-  2.07 | 22.66  +/-  2.64 | 0.886 |
| **Direct social interaction test** |  |  |  |  |  |  |  |  |  |
| Latency to the first proximity | 3.17  +/-  0.96 | 4.30  +/-  1.52 | > 0.999 | 7.76  +/-  4.04 | 21.03  +/-  11.71 | 0.190 | 12.11  +/-  4.77 | 10.41  +/-  3.11 | > 0.999 |
| Proximity counts | 54.62  +/-  6.36 | 52.00  +/-  5.03 | > 0.999 | 43.67  +/-  14.01 | 32.83  +/-  4.56 | > 0.999 | 27.71  +/-  2.83 | 32.09  +/-  4.20 | > 0.999 |
| Cumulative duration percentage | 36.87  +/-  3.37 | 36.42  +/-  3.84 | > 0.999 | 31.61  +/-  2.71 | 30.50  +/-  3.21 | > 0.999 | 34.93  +/-  3.82 | 40.62  +/-  3.22 | 0.855 |
| **Fear conditioning** |  |  |  |  |  |  |  |  |  |
| Percentage of freezing (Acquisition) | 24.86  +/-  3.65 | 36.14  +/-  2.33 | 0.216 | 24.17  +/-  7.16 | 30.33  +/-  8.47 | > 0.999 | 2.57  +/-  0.57 | 2.13  +/-  0.88 | > 0.999 |
| Percentage of freezing (Context memory) | 37.43  +/-  6.80 | 76.29  +/-  8.33 | < 0.0001 | 23.83  +/-  8.68 | 21.83  +/-  3.89 | > 0.999 | 4.29  +/-  1.25 | 4.63  +/-  1.18 | > 0.999 |
| Percentage of freezing (Cued memory) | 27.71  +/-  8.81 | 51.29  +/-  7.53 | 0.009 | 11.83  +/-  3.32 | 11.83  +/-  4.73 | > 0.999 | 3.71  +/-  1.15 | 2.88  +/-  0.72 | > 0.999 |
| **Active place avoidance test** |  |  |  |  |  |  |  |  |  |
| Latency to enter the shock area after 24 hrs | 343.48  +/-  100.99 | 150.37  +/-  90.50 | 0.062 | 12.97  +/-  9.34 | 29.42  +/-  7.82 | > 0.999 | 22.03  +/-  6.01 | 24.24  +/-  12.56 | > 0.999 |
| Number of shocks after 24 hrs | 28.17  +/-  15.23 | 34.83  +/-  9.24 | > 0.999 | 74.50  +/-  13.25 | 57.83  +/-  9.65 | 0.952 | 82.43  +/-  9.27 | 82.80  +/-  12.21 | > 0.999 |

**Supplementary Table 2: Comparison between male and female mice of C57BL/6N, DBA/2, and FVB/N strains in the LABORAS test at P46**

| **Name of Test** | **C57BL/6N** |  |  | **DBA/2** |  |  | **FVB/N** |  |  |
| --- | --- | --- | --- | --- | --- | --- | --- | --- | --- |
|  | ♂ Mean +/- SEM | ♀ Mean +/- SEM | Bonferroni test | ♂ Mean +/- SEM | ♀ Mean +/- SEM | Bonferroni test | ♂ Mean +/- SEM | ♀ Mean +/- SEM | Bonferroni test |
| **LABORAS** |  |  |  |  |  |  |  |  |  |
| Locomotion duration | 61.68  +/-  6.65 | 61.87  +/-  8.73 | > 0.999 | 37.26  +/-  1.65 | 37.09  +/-  3.54 | > 0.999 | 105.44  +/-  10.01 | 86.87  +/-  5.34 | 0.152 |
| Distance | 392.49  +/-  33.86 | 392.57  +/-  33.78 | > 0.999 | 203.00  +/-  17.42 | 153.33  +/-  13.02 | 0.518 | 491.48  +/-  31.27 | 386.35  +/-  19.82 | 0.016 |
| Average speed | 4.54  +/-  0.39 | 4.54  +/-  0.39 | > 0.999 | 2.35  +/-  0.20 | 1.77  +/-  0.15 | 0.518 | 5.69  +/-  0.36 | 4.47  +/-  0.23 | 0.016 |
| Maximum speed | 192.54  +/-  7.58 | 205.30  +/-  2.26 | > 0.999 | 279.25  +/-  10.83 | 270.67  +/-  9.59 | > 0.999 | 336.00  +/-  15.54 | 292.38  +/-  14.00 | 0.024 |
| Rearing counts | 1517.13  +/-  122.93 | 1257.00  +/-  111.44 | > 0.999 | 1125.75  +/-  77.97 | 856.33  +/-  135.83 | 0.918 | 2871.44  +/-  274.34 | 2496.38  +/-  262.79 | 0.470 |
| Self-grooming counts | 600.50  +/-  86.30 | 684.57  +/-  74.55 | 0.871 | 542.00  +/-  30.94 | 628.11  +/-  49.18 | 0.748 | 412.89  +/-  28.22 | 425.88  +/-  31.27 | > 0.999 |
| Climbing counts | 158.63  +/-  25.34 | 205.86  +/-  48.80 | > 0.999 | 356.13  +/-  50.91 | 550.00  +/-  95.98 | 0.457 | 1089.44  +/-  164.28 | 742.75  +/-  77.67 | 0.038 |
| Eating counts | 1339.00  +/-  119.64 | 1528.14  +/-  73.11 | 0.555 | 881.63  +/-  91.07 | 701.89  +/-  98.27 | 0.539 | 1909.11  +/-  81.87 | 1433.75  +/-  94.07 | 0.002 |
| Drinking counts | 113.13  +/-  16.30 | 108.43  +/-  27.55 | > 0.999 | 13.38  +/-  3.20 | 10.22  +/-  2.29 | > 0.999 | 21.33  +/-  3.60 | 16.13  +/-  6.95 | > 0.999 |
